# The Involvement of Temporomandibular Joint in Psoriatic Arthritis: A Report of a Rare Case

**DOI:** 10.7759/cureus.20392

**Published:** 2021-12-13

**Authors:** Roopa R, T Malarkodi, Emmanuel Azariah, Aravind S Warrier

**Affiliations:** 1 Oral Medicine and Radiology, Sri Ramachandra Faculty of Dental Sciences, Sri Ramachandra Institute of Higher Education and Research, Chennai, IND; 2 Oral Medicine and Radiology, Sri Ramachandra Institute of Higher Education and Research, Chennai, IND; 3 Oral and Maxillofacial Surgery, Sri Ramachandra Institute of Higher Education and Research, Chennai, IND

**Keywords:** caspar criteria, human leukocyte antigen b27, psoriasis, temporomandibular joint, psoriatic arthritis

## Abstract

Psoriatic arthritis (PsA) is a chronic inflammatory condition affecting psoriatic patients. Its clinical manifestations in patients can vary over time, advancing from one joint to the next with an intermittent pattern of exacerbation and remission. The condition shares similar manifestations with rheumatoid arthritis (RA), ankylosing spondylitis, and reactive arthritis; hence, a comprehensive examination is required for a proper diagnosis and management. It is associated with an increased risk of comorbidities affecting patients' well-being. There have been few incidences of involvement extending to the temporomandibular joint (TMJ), but a proper record needs to be maintained to evaluate its part in PsA. In this report, we present a case of PsA in which the patient complained of ear pain and was discovered to have early alterations in the TMJ.

## Introduction

Psoriatic arthritis (PsA) is a musculoskeletal inflammatory condition that affects psoriatic patients [1]. Psoriasis is an autoimmune disease characterized by erythematous plaque formation in the extensor surfaces, lumbosacral area, and a silvery scale-covered scalp [2]. In 5-42% of psoriatic patients, PsA is associated with both inflammatory and autoimmune qualities [1,3,4]. Different clinical manifestations can appear over time, advancing from one joint to the next with an intermittent pattern of exacerbation and remission [4,5]. The involvement of the temporomandibular joint (TMJ) is said to be unusual, despite the fact that it might affect other joints [6]. We present a case of PsA in a patient who complained of ear pain and was later found to have early alterations in the TMJ.

## Case presentation

A 44-year-old presented with a complaint of right-ear pain for one month. The discomfort was sporadic, sharp-pricking in character, worsened during mouth opening, and non-radiating. For the past 20 years, he had been treated for psoriasis with methotrexate 25 mg, folic acid 5 mg, and naproxen 500 mg. The patient had also been experiencing joint pain and stiffness in his fingers, toes, knees, cervical vertebrae, and neck for the past seven years. 

**Figure 1:**
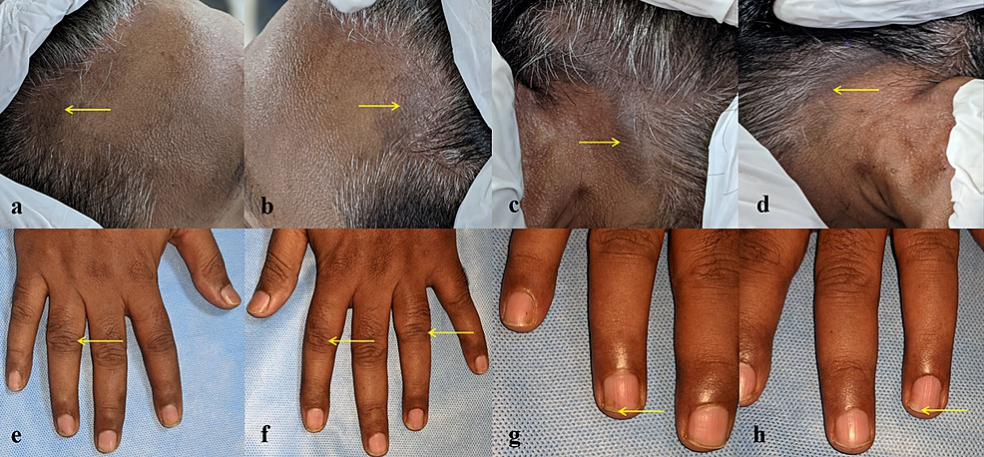
General examination findings Yellow arrows indicate hyperpigmentation in the hairline region of the forehead (a, b), behind the right and left ears (c, d), dactylitis (e, f), and onycholysis (g, h)

Hyperpigmentation was found in the hairline region of the forehead, behind the ears, and in the occipital region during a general examination. There were signs of onycholysis, dactylitis, spine restriction, and neck pain with stiffness (Figure 1).

**Figure 2:**
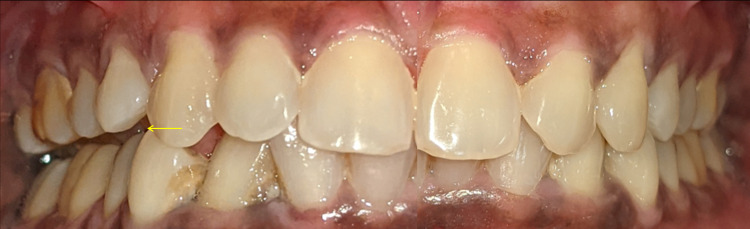
Intraoral image showing derangement of occlusion (yellow arrow) on the right side

Extraoral examination revealed tenderness over the right TMJ region while opening, as well as lateral and forward jaw movements. There was no clicking or deviation while opening and closing the jaw. Palpation revealed tenderness over the right and left lateral pterygoid muscles. When opening and closing the mouth, there was evidence of crepitus in the right and left TMJ and the interincisal distance was 38 mm. Intraoral examination revealed right-sided occlusal derangement, no midline shift, and partially erupted teeth 38, 48 (Figure 2).

PsA with TMJ involvement (TMJ PsA) was suspected based on the history and clinical presentation. The patient was referred to a rheumatologist after obtaining a panoramic radiograph and a cone-beam CT (CBCT) scan of the TMJ. Tenderness in the right TMJ was found during the rheumatologist's examination. Cervical spine flexion and lateral rotation were both within the range of motion; there was no other active arthritis, and bilateral Faber's tests were negative. The rheumatologist diagnosed PsA based on the patient's history and clinical features and recommended a basic Rh profile, arthritis profile, human leukocyte antigen B27 (HLA-B27) flow cytometry (Table 1), and MRI of both the sacroiliac (SI) joints with whole spine screening to rule out rheumatoid arthritis (RA).

**Table 1:** Blood investigation report ESR: erythrocyte sedimentation rate; CRP: c-reactive protein; RF test: rheumatoid factor test; anti-CCP: anti-cyclic citrullinated peptide; HLA-B27: human leukocyte antigen B27

Investigations	Values
ESR	18 mm/hr
CRP	0.9 mg/dL
RF test	20.0 IU/ml
Uric acid	6.9 mg/dL
Anti-CCP	7.0 U/mL
HLA-B27 flow cytometry	Negative

**Figure 3:**
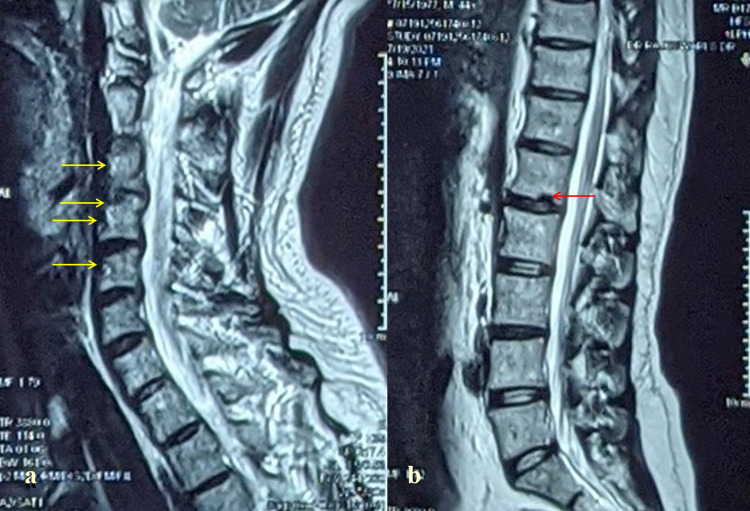
T2-weighted MRI (sagittal view) of whole spine showing early degenerative changes in the cervical (yellow arrow) (a) and lumbar (red arrow) spine (b) MRI: magnetic resonance imaging

MRI whole spine screening demonstrated early degenerative changes in the cervical and lumbar spine. There was no ensuing neuronal compression (Figure 3).

**Figure 4:**
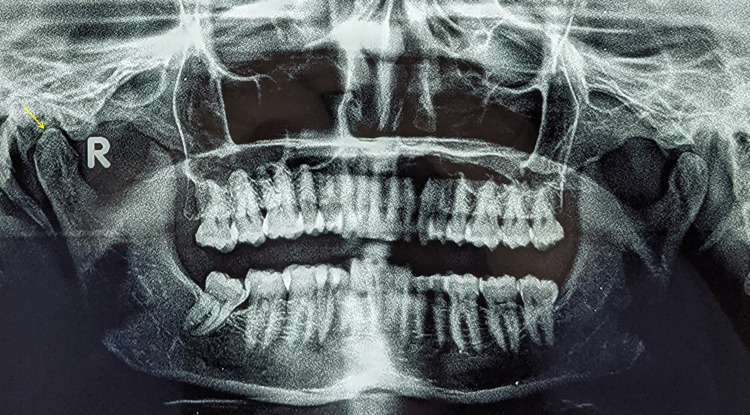
Panoramic radiograph showing mild bony changes (yellow arrow) in the right condyle

**Figure 5:**
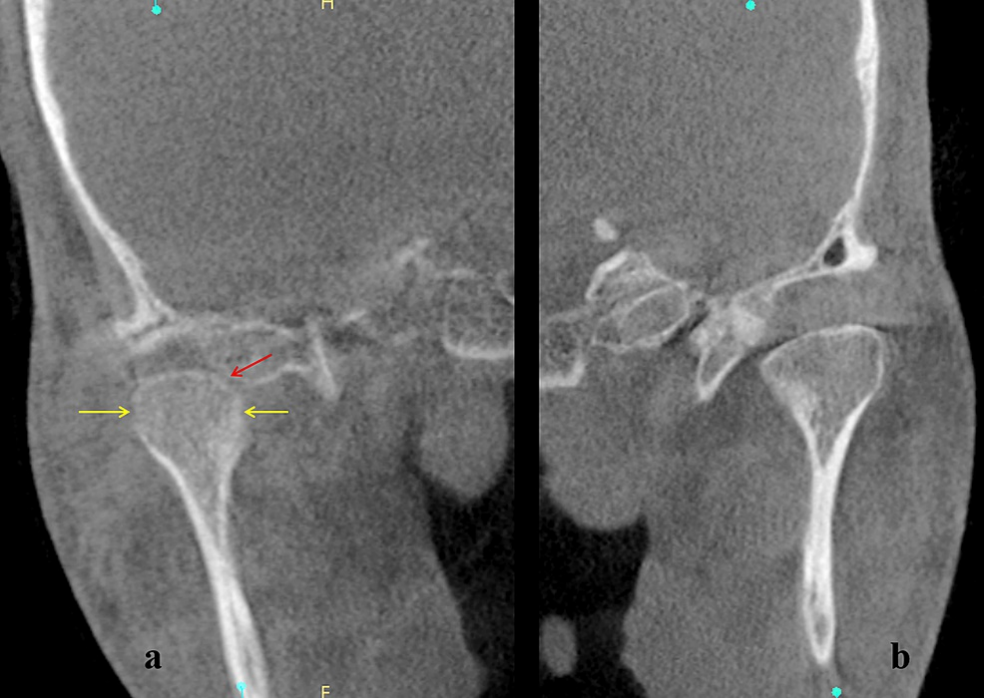
CBCT showing mild bone erosion (yellow arrow) in the medial and lateral aspects of the right condyle with a reduction in articular space (red arrow) (a), normal left condyle (b) CBCT: cone-beam computed tomography

The panoramic radiograph showed mild bony changes in the right condyle (Figure 4). Since a panoramic radiograph is not an optimal choice for the evaluation of the articular space, a CBCT scan was done. CBCT of the TMJ revealed mild bone erosion in the medial and lateral aspects of the right condyle, as well as a reduction in articular disc space on the right side, indicating early degenerative changes in the right condyle (Figure 5).

Prednisolone 10 mg once daily, ranitidine 150 mg twice daily, calcium with vitamin D3 (Calten D) once daily for seven days, methotrexate 15 mg once a week (Fridays), and folic acid 5 mg once daily (except Fridays) for seven weeks were prescribed orally for the patient. The patient has been advised a soft diet and minimal opening (range of movements) of the mouth. He has also been advised to have all third molars extracted followed by splint therapy.

## Discussion

PsA is a chronic musculoskeletal disease characterized by psoriasis and joint inflammation [7]. It has comparable clinical characteristics with spondyloarthropathies and RA [3]. The fingers, spine, and nails are the most frequently affected areas in PsA. Both genders are equally affected, and the majority of cases are seen in people between the ages of 40 and 50 years [8]. There is an ~6 in 100,000 incidence rate per year and ~1-2 in 1,000 prevalence of PsA in the general population. And in psoriasis patients, the incidence of PsA is 2.7% annually and the prevalence varies between 6 and 41% [9]. Systemic disorders such as hypertension, dyslipidemia, cardiovascular disease, diabetes, and obesity are more closely linked with this condition, and the leading cause of morbidity and mortality is cardiovascular disease [8].

Genetic factors have a significant role in the development of PsA, and they are found in more than 80% of people with the disease. The strongest genetic risk factors include variations in the HLA-B and HLA-C genes, but more than 30 additional variants also play a role. Many of these variations are similar to those linked to psoriasis, which has over 40 susceptibility loci. These genes are found near or within the signaling pathways for interleukin (IL)-12, IL-23, and nuclear factor kappa B (NFB). The condition is believed to be induced by an environmental trigger, most likely infectious in nature, which causes immunological activation involving dendritic cells and T cells in genetically susceptible individuals. Within the joint, CD8+ T cells (which identifies antigen presented in the context of HLA class I) outnumber CD4+ T cells, which is consistent with the genetic link between PsA and HLA-C and B variations. The IL-23/IL-17 pathway appears to play a key role in PsA, according to mounting evidence. The triggering event is thought to drive dendritic cells to overproduce IL-23, which promotes the differentiation and activation of Th17 cells, which produce the pro-inflammatory cytokine IL-17A. At entheses, in the bone, and within the joint, this, along with Th1 cytokines like interferon (IFN)-gamma and tumor necrosis factor (TNF)-alpha, acts on macrophages and tissue-resident stromal cells to create more pro-inflammatory cytokines and other mediators, which contribute to inflammation and tissue damage [3,10,11].

Pain, stiffness of joints, spine, tendons, and entheses are common symptoms of the condition. In most cases, the joints are not swollen. Patients with PsA may experience articular, articular/periarticular, and extra-articular symptoms. Oligoarticular arthritis, polyarticular arthritis, distal arthritis, arthritis mutilans, and spondyloarthritis are some of the articular manifestations. Peripheral arthritis, either oligoarticular or polyarticular arthritis, periarticular diseases, such as enthesitis, dactylitis, tenosynovitis, and axial arthritis are all examples of articular/periarticular manifestations. Cutaneous psoriasis, nail disease such as pitting, onycholysis, and splinter hemorrhages, and ocular disease such as uveitis are examples of extra-articular presentations [3,12,13].

Pain, swelling over the pre-auricular region, jaw deviation while opening/closing, occlusal derangement, inability to adequately open or close the mouth, and reduced mouth opening are all manifestations of TMJ involvement [14]. The classification criteria for psoriatic arthritis (CASPAR) 2006 are used to diagnose PsA, and the patient must have 3 points to be diagnosed. CASPAR criteria include (i) 2 points if the patients have skin psoriasis, 1 point if they previously had it, or 1 point if it runs in the family but the patient is unaffected, (ii) 1 point for nail lesions such as pitting, onycholysis, and hyperkeratosis, (iii) 1 point if dactylitis was previously or is currently present, (iv) 1 point in case of negative rheumatoid factor (RF) as investigated by any method except by latex, and (v) patients should have juxta-articular bone formation. And these criteria are said to have 91% sensitivity and 99% specificity. Other criteria that were used previously are Moll and Wright criteria, Bennett criteria, Vasey and Espinoza criteria, the European Spondyloarthropathy Study Group (ESSG) criteria, Fournie criteria, and the McGonagle criteria [4,15].

Since the clinical manifestations of PsA are similar to those of RA, ankylosing spondylitis, and reactive arthritis, a thorough examination is essential to make a diagnosis. PsA is distinguished from reactive arthritis by the presence of cutaneous psoriasis, concomitant nail signs, and polyarthritis affecting the distal interphalangeal (DIP) joints. Reactive arthritis is characterized by polyarthritis affecting small joints and keratoderma blennorrhagica. Ankylosing spondylitis and PsA have a few similarities in terms of symptoms. Peripheral arthritis involving large joints, symmetric sacroiliitis, and continuous spondylitis with delicate marginal syndesmophytes distinguish ankylosing spondylitis from PsA, which has asymmetric sacroiliitis and discontinuous spondylitis with bulky non-marginal syndesmophytes. Polyarthritis affects the finger joints, particularly metacarpophalangeal (MCP) joints and proximal interphalangeal (PIP) joints, wrists, and the presence of subcutaneous nodules, vasculitis, scleritis, episcleritis, and cervical-spine inflammation with no involvement of the sacroiliac joints differentiates RA from PsA. PsA, on the other hand, will have uveitis, as well as symmetric or asymmetric polyarthritis affecting DIP joints and asymmetric involvement of the sacroiliac joint, as previously mentioned [3,12,16].

In these circumstances, RF, anti-CCP, and HLA-B27 tests are performed. RA tests positive for both RF and anti-CCP but is not linked to HLA-B27. Ankylosing spondylitis and reactive arthritis are both negative for RF and anti-CCP, with the majority of cases positive for HLA-B27. PsA is usually negative for both RF and anti-CCP, with exception of axial disease, which is HLA-B27-positive [3].

The presence of the triad of psoriasis, erosive polyarthritis, and a negative RF serological test leads to the diagnosis. Our patient had healing cutaneous psoriasis lesions, nail involvement, peripheral arthritis involving the small joints, neck pain with stiffness, cervical and lumbar spine degeneration with occlusal derangement, TMJ degeneration, and negative RF and anti-CCP, as well as negative HLA-B27, all of which confirmed the diagnosis of TMJ PsA. Although other imaging modalities like CT, CBCT, and orthopantomography (OPG) can be used to assess inflammation, MRI and ultrasonography (USG) are the best options [17,18].

The first and most important step in treating TMJ symptoms is to alleviate pain. Conservative care, which includes a soft diet, minimum mouth opening, warm fomentation over the muscle spasm, non-steroidal anti-inflammatory drugs (NSAIDs), physiotherapy, and an occlusal splint, usually resolves symptoms in 80% of the cases. Occlusal splints relieve myofascial pain by allowing the masticatory muscle to rest in its passive positive position, which relieves pressure on the TMJ. Although a local anesthetic (lignocaine 1% or 2%) can be infused into the joint region, the discomfort disappears after 10 minutes; 0.5% bupivacaine can be injected into the temporalis and masseter to relieve myofascial pain and spasms momentarily. Botulinum toxin can also be administered; however, it produces muscle paralysis that can last up to six months and only reduces pain by 25%. TMJ synovitis is relieved by intra-articular steroid injection. However, it has been shown on MRI and during arthroscopy that it can cause joint inflammation and cartilage thinning, and hence it is not widely recommended. Ohnishi was the first to describe TMJ arthroscopy with arthrocentesis in 1975, which aids in diagnostic and treatment planning, flushes out debris, lavages the joint under pressure with saline, and breaks down adhesions that inhibit normal movement. Usually, this procedure is well-tolerated, minimally invasive, low-cost, and can be performed even under local anesthesia with minimal risk of degenerative changes. There is an even better outcome when these two procedures are combined, with improvement in mouth opening. These treatments can be supplemented by disease-modifying antirheumatic drugs (DMARDs) to lower disease activity and inflammation [14].

## Conclusions

Psoriasis is rarely known to involve TMJ, which causes an increase in orofacial pain. Because of the ambiguous nature of the symptoms associated with PsA, a thorough case history, clinical examination, as well as laboratory and radiographic studies are required. PsA affecting TMJ is often underrecognized or undertreated. Delayed diagnosis will lead to extensive destruction of the TMJ structures causing chronic pain and affecting the quality of the life. Hence, a consistent diagnostic approach must be implemented for cutaneous psoriasis associated with TMJ arthritis and necessary treatment must be initiated at the earliest to improve patients' well-being.
